# Effect of Virtual Reality on Anxiety, Pain, and Radial Artery Spasm During Transradial Coronary Angiography: A Prospective Randomized Controlled Trial

**DOI:** 10.3390/jcm15187227

**Published:** 2026-09-17

**Authors:** Ahmet Burak Keçeci, Emrah Yeşil

**Affiliations:** 1 Department of Cardiology, Tarsus State Hospital, 33460 Mersin, Türkiye; aburakkececi@gmail.com; 2 Department of Cardiology, Faculty of Medicine, Mersin University, 33343 Mersin, Türkiye

**Keywords:** transradial coronary angiography, radial artery spasm, virtual reality, anxiety, pain

## Abstract

**Background**: Radial artery spasm (RAS) is a major complication of transradial coronary angiography (TRA) that adversely affects procedural success and patient comfort. Although procedural anxiety and pain contribute to RAS development, prospective randomized controlled data on the effect of virtual reality (VR) on RAS are limited. This study aimed to evaluate the effects of VR on anxiety, pain, and RAS during TRA. **Methods**: In this prospective, single-center, randomized controlled trial, 236 patients undergoing elective TRA were randomized 1:1 to the VR or control group. Following prespecified post-randomization exclusions, 130 patients (65 per group) were included in the per-protocol analysis. The intervention group received standardized VR content throughout the procedure. The primary endpoint was clinically assessed RAS; secondary endpoints included pre- and post-procedural state anxiety (STAI-S) and post-procedural pain measured using the visual analog scale (VAS). **Results**: Clinically assessed RAS occurred less frequently in the VR group than in the control group (7.7% vs. 52.3%, *p* < 0.001). Post-procedural STAI-S and VAS scores were also lower in the VR group (both *p* < 0.001). In an exploratory multivariable model, post-procedural STAI-S (OR, 1.29; 95% CI, 1.13–1.47; *p* < 0.001) and VAS scores (OR, 3.44; 95% CI, 1.98–5.98; *p* < 0.001) were associated with RAS. Because these variables were measured after the procedure, temporal direction and mediation cannot be inferred. **Conclusions**: In this single-center per-protocol analysis, VR use was associated with lower post-procedural anxiety and pain and a lower incidence of clinically assessed RAS during TRA. The large effect estimate should be interpreted cautiously because the intervention could not be blinded, the endpoint included subjective clinical features, and substantial post-randomization exclusions limit generalizability. Multicenter trials with blinded or objective endpoint corroboration and intention-to-treat analyses are warranted.

## 1. Introduction

Transradial coronary angiography (TRA) has become the standard vascular access for diagnostic coronary angiography because of its lower rates of bleeding and vascular complications, earlier mobilization, and greater patient comfort than transfemoral access [1,2,3].

Despite these advantages, radial artery spasm (RAS) remains one of the most common complications of TRA. Previous studies have reported a wide range of RAS incidence, reaching up to 51.3%, depending on the diagnostic criteria, patient population, premedication strategies, and sheath and catheter characteristics. Although the routine use of hydrophilic sheaths and intra-arterial vasodilators has reduced its incidence, RAS remains an important complication that may cause patient discomfort, hinder catheter manipulation, prolong the procedure, increase radiation exposure, and occasionally require crossover to an alternative vascular access. Furthermore, vasodilators commonly used to prevent and treat RAS may cause hemodynamic adverse effects such as hypotension and bradycardia, limiting their use, particularly in susceptible patients [4,5,6,7,8,9,10].

The pathophysiology of RAS is multifactorial and involves mechanical, anatomical, pharmacological, and psychological factors. Female sex, smaller radial artery diameter, repeated catheter manipulation, and inadequate vasodilator use have been associated with RAS [4,5,8,9,10]. Procedural anxiety has also been reported as an associated factor [11,12], with autonomic activation, vascular tone, and pain perception representing biologically plausible—but untested in this trial—links [13,14]. Accordingly, non-pharmacological approaches to procedural anxiety have received increasing attention [15,16].

Virtual reality (VR) has emerged as a promising non-pharmacological intervention for reducing anxiety across a wide range of medical settings. Through its immersive audiovisual distraction, VR can reduce patients’ awareness of external stimuli, attenuate stress responses, and improve tolerance to medical procedures. Recent studies have demonstrated that VR effectively reduces procedure-related anxiety and pain during various invasive interventions, including endoscopic procedures, dental interventions, and cardiovascular procedures. Notably, a pilot study conducted during transcatheter aortic valve implantation (TAVI) demonstrated that VR significantly reduced patients’ anxiety levels. Similarly, another study involving coronary angiography reported that VR improved patient satisfaction while reducing procedure-related anxiety [17,18,19,20,21,22,23,24,25]. Given the potential role of anxiety in the pathogenesis of RAS, VR may represent a practical non-pharmacological strategy for mitigating RAS by alleviating procedure-related anxiety. However, despite its established anxiolytic effects, the impact of VR on the development of RAS during TRA has not yet been adequately investigated.

Therefore, this randomized trial evaluated whether VR-based distraction during TRA was associated with differences in clinically assessed RAS, procedure-related anxiety, pain perception, and treatment-requiring RAS. The trial was not designed to establish a mechanistic mediation pathway between anxiety, pain, and RAS.

## 2. Materials and Methods

### 2.1. Study Design and Study Population

This prospective, single-center, randomized controlled trial was conducted in the Coronary Angiography Unit of Mersin University Faculty of Medicine between January and July 2025.

The study protocol was approved by the Mersin University Clinical Research Ethics Committee (7 August 2024; Approval No. 2024/752) and was conducted in accordance with the principles of the Declaration of Helsinki. Permission to use the study questionnaires was obtained from the respective copyright holders, and written informed consent was obtained from all participants before enrollment. The trial was prospectively registered at ClinicalTrials.gov (https://clinicaltrials.gov/study/NCT06726031 Identifier: NCT06726031; registered on 10 December 2024, accessed on 15 September 2026).

Patients aged 18 years or older who were scheduled to undergo elective transradial access (TRA), had no previous history of coronary angiography, were conscious, oriented, and cooperative, were able to read and understand Turkish, had a palpable radial pulse, and provided written informed consent were eligible for inclusion.

Patients were excluded if they had an indication for coronary angiography due to acute coronary syndrome, were scheduled to undergo a non-transradial access procedure, were unable to cooperate, had a physical condition that precluded the use of a VR headset, had a history of neuropsychiatric disease, anxiety disorder, or treatment for these conditions, had a history of malignancy, or had evidence of impaired upper extremity circulation.

**Figure 1:**
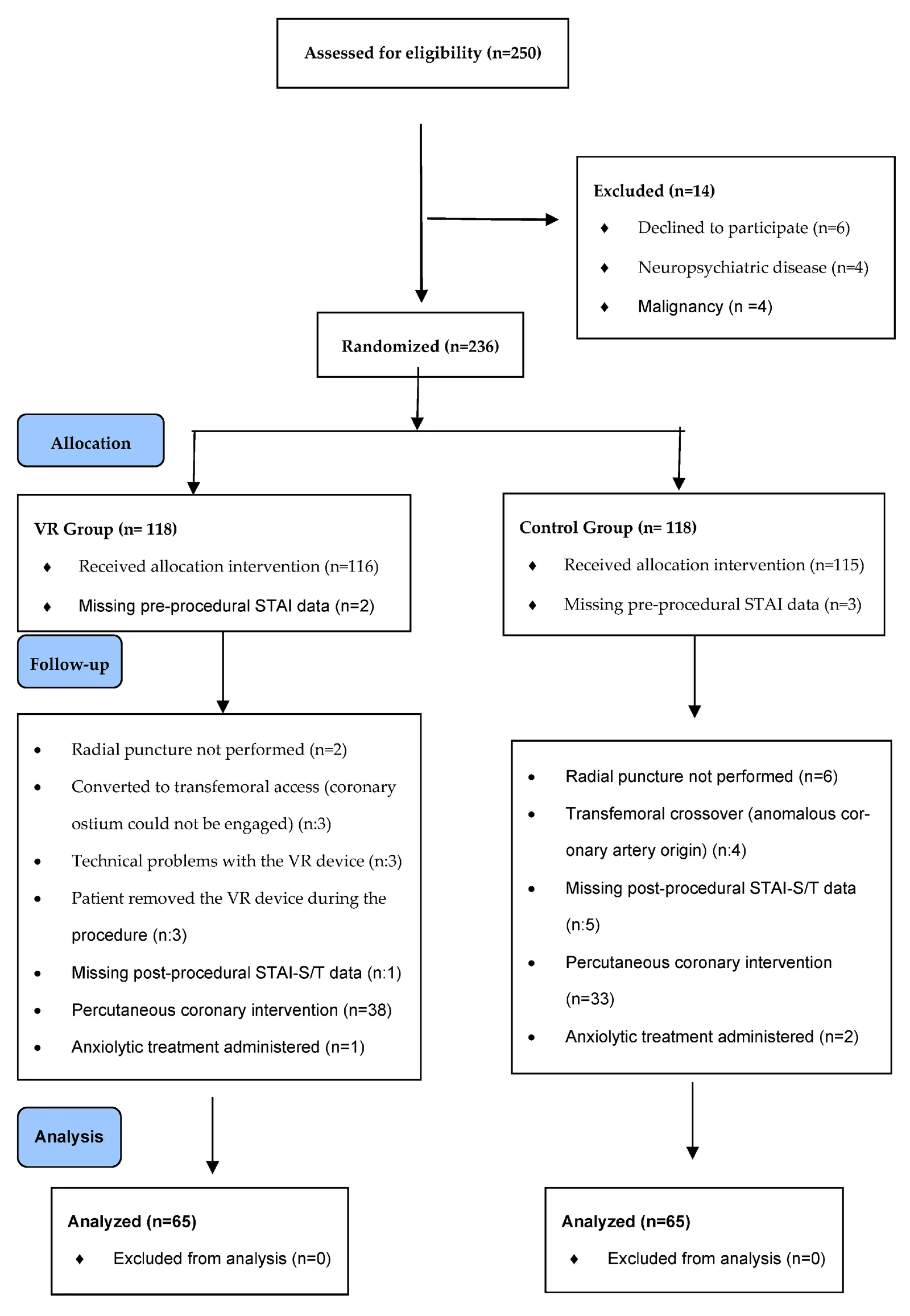
Consort Flow Diagram.

Patient selection, randomization, post-randomization exclusions, and the per-protocol analysis set are summarized in the CONSORT flow diagram (Figure 1). Because patients who underwent PCI and those meeting other prespecified post-randomization exclusion criteria were not included in the final analysis, the analysis was not intention-to-treat.

In the absence of sufficient preliminary data on the effect of VR on RAS, the planned sample size was estimated from a pilot study of 20 participants (10 per group). A post hoc calculation based on the final sample of 130 participants indicated 99% power for the observed between-group difference; this retrospective estimate is descriptive and does not address bias or precision arising from post-randomization exclusions.

### 2.2. Randomization and Study Procedures

Eligible patients were randomly assigned at a 1:1 ratio to either the VR intervention group or the control group using a computer-generated simple randomization sequence. Allocation concealment was ensured through sequentially numbered, opaque, sealed envelopes prepared by an independent researcher who was not involved in patient recruitment or study procedures.

Because the VR headset was visible, participants and the treating operator could not be blinded after allocation; the trial was therefore open-label. Allocation remained concealed until assignment, and data analysis was performed by an independent statistician blinded to group allocation. RAS was assessed by the unblinded operator using the prespecified clinical criteria below; no independent blinded adjudication, angiographic confirmation, or ultrasound corroboration was used. This limitation is relevant because several diagnostic components were subjective.

Venous blood samples for laboratory analyses were collected before TRA while patients were resting supine. Baseline chronic medication use was recorded at enrollment and is reported in Table 1. TSH was measured using an immunoenzymatic sandwich assay on the DxI 800 Immunoassay System (Beckman Coulter Inc., Brea, CA, USA), and conventional CRP was measured using an immunoturbidimetric assay on the AU5800/AU680 Clinical Chemistry Analyzer (Beckman Coulter Inc., Brea, CA, USA). Other biochemical parameters were measured in the central laboratory using routine validated methods.

All TRA procedures were performed through the right radial artery by one experienced interventional cardiologist. A 6 Fr hydrophilic radial sheath was used uniformly because this was the institution’s standard diagnostic platform during the study period and standardized sheath-related mechanical exposure across groups; 5 Fr systems were not evaluated. After sheath placement, every patient received 5000 IU unfractionated heparin and 100 μg intra-arterial nitroglycerin. CCBs were not administered routinely and were reserved for treatment of RAS. Diagnostic angiography used standard 6 Fr Judkins catheters (Boston Scientific Corporation, Marlborough, MA, USA) and a nonionic, low-osmolality iohexol-based contrast agent.

Procedural characteristics, including procedure duration, fluoroscopy time, radiation exposure, contrast volume, and the number of puncture attempts, were prospectively recorded. Data analysis was performed by an independent statistician who was blinded to group allocation.

### 2.3. Virtual Reality Intervention

Patients assigned to VR used an Oculus Meta Quest 3 headset (Meta Platforms Inc., Menlo Park, CA, USA) displaying the same nature-based visual environments with nonverbal background music. After sterile draping, a trained study team member positioned and secured the headset at the patient’s head, outside the sterile right-radial access field, and initiated the content before local anesthesia. The headset did not cross or contact the sterile field. The team member monitored fit, audiovisual continuity, patient communication, and tolerance without interfering with the operator or sterile equipment. The headset remained in place throughout angiography unless the patient requested removal because of discomfort or a technical interruption; the protocol did not systematically record specific symptoms such as claustrophobia or vertigo among removals. The intervention ended after sheath removal and compression-band placement. The procedural arrangement of the patient, healthcare personnel, VR equipment, angiography monitor, and sterile field is illustrated schematically in Supplementary Figure S1.

Patients assigned to the control group underwent the procedure according to the institution’s standard clinical practice without any visual or auditory distraction intervention.

### 2.4. Definition of Radial Artery Spasm

RAS was operationally diagnosed using the five-item clinical criteria described by Ercan et al. [11], which were selected before enrollment: at least two of (1) persistent forearm pain; (2) pain during catheter manipulation; (3) pain during sheath withdrawal; (4) difficulty advancing the catheter or a sensation of catheter entrapment; and (5) resistance during sheath removal. Related studies using clinical RAS definitions are cited for context [4,26,27,28,29].

In addition to the overall incidence of RAS, treatment-requiring RAS was prospectively assessed. Treatment-requiring RAS was defined as spasm severe enough to interfere with procedural progress and necessitate additional non-pharmacological and/or pharmacological interventions beyond routine procedural management. Non-pharmacological measures included temporary cessation of catheter manipulation, verbal reassurance, local heat application, the Balbay maneuver, and optimization of arm positioning [30,31]. Pharmacological treatment was administered in a stepwise manner according to the study protocol. In patients who did not respond adequately to non-pharmacological measures and were hemodynamically stable, 200 μg of intra-arterial nitroglycerin and/or 5 mg of diltiazem were administered [32,33,34]. When spasm persisted, 100 μg of fentanyl was administered to improve pain control and procedural tolerance [35,36]. In refractory cases, midazolam was administered as anxiolytic therapy [35,37]. Patients who received midazolam were excluded from the study analysis because of its potential to influence anxiety assessments. If RAS persisted despite all pharmacological and non-pharmacological interventions, the procedure was terminated or an alternative vascular access site was used.

### 2.5. Assessment of Anxiety and Pain

State and trait anxiety were assessed with the State–Trait Anxiety Inventory (STAI). STAI-S and STAI-T were completed before the procedure and again after radial sheath removal while the patient rested in bed. STAI-S ranges from 20 to 80, with higher scores indicating greater state anxiety [38,39]. The post-procedural timing overlapped the period after RAS occurrence and any required treatment; therefore, post-procedural scores were treated as correlates, not temporally antecedent predictors or mediators.

Procedure-related changes in state anxiety (ΔSTAI-S) were calculated as the difference between the pre-procedural and post-procedural STAI-S scores (pre-procedural STAI-S − post-procedural STAI-S). Positive values indicated a reduction in state anxiety during the procedure, whereas negative values indicated an increase in anxiety.

Procedure-related pain intensity was assessed after radial sheath removal using a 10-point Visual Analog Scale (VAS). Higher VAS scores indicated greater pain intensity [40].

All questionnaires and assessment forms were completed by the participants under the supervision of the study personnel in accordance with the standardized study protocol.

### 2.6. Study Endpoints

The primary endpoint of the study was the incidence of RAS during TRA.

Secondary endpoints included the post-procedural STAI-S score, VAS score, ΔSTAI-S, procedure duration, fluoroscopy time, radiation exposure, contrast volume, and the number of puncture attempts. Among patients who developed RAS, the need for additional interventions was also evaluated.

Because the diagnosis of RAS was based on previously validated clinical criteria with high sensitivity, treatment-requiring RAS was analyzed as a separate endpoint to distinguish more clinically significant spasm events requiring active intervention.

### 2.7. Statistical Analysis

All statistical analyses were performed using IBM SPSS Statistics for Windows, Version 25.0 (IBM Corp., Armonk, NY, USA). The normality of continuous variables was assessed using the Shapiro–Wilk test. Normally distributed continuous variables are presented as mean ± standard deviation (SD), whereas non-normally distributed variables are presented as median (interquartile range [IQR]) and minimum–maximum values. Categorical variables are expressed as frequencies and percentages.

Categorical variables were compared using the chi-square test or Fisher’s exact test, as appropriate. Continuous variables were compared using the independent-samples Student’s *t* test for normally distributed data and the Mann–Whitney *U* test for non-normally distributed data. Paired comparisons between pre-procedural and post-procedural measurements were performed using the paired *t* test for normally distributed variables and the Wilcoxon signed-rank test for non-normally distributed variables.

Variables associated with RAS in univariable analysis (*p* < 0.05) were entered into an exploratory multivariable binary logistic regression model. Results are reported as odds ratios (ORs) with 95% confidence intervals (CIs). Because VAS and post-procedural STAI-S were measured after the procedure and after possible RAS/treatment, their model coefficients describe cross-sectional associations and cannot establish prediction, causal direction, or mediation.

Analyses restricted to patients with RAS were exploratory. Post-procedural STAI-S, ΔSTAI-S, and VAS were summarized by VR use and treatment strategy. Inferential testing was performed only where group sizes allowed; very small VR subgroups were described without formal testing. Significant omnibus tests were followed by Tukey or Dunn pairwise comparisons, as appropriate.

All statistical tests were two-sided, and a *p* value < 0.05 was considered statistically significant. All subgroup analyses were prespecified before database lock and conducted according to the study protocol.

## 3. Results

A total of 250 patients were assessed for eligibility. Of these, 14 were excluded before randomization because they did not meet the eligibility criteria or declined to participate. The remaining 236 patients were randomly assigned in a 1:1 ratio to either the VR intervention group (n = 118) or the control group (n = 118).

Following prespecified post-randomization exclusions, including exclusion of patients who underwent PCI, 65 participants in each group were retained in the per-protocol analysis (130/236 randomized; 55.1%). Thus, 106 randomized participants (44.9%) were not represented in the primary analysis. The numbers and reasons by group are shown in Figure 1.

Baseline demographic, clinical, laboratory, and echocardiographic characteristics of the study population are presented in Table 1. Overall, the VR and control groups were well balanced with respect to demographic characteristics, cardiovascular risk factors, baseline medications, laboratory findings, and left ventricular ejection fraction. However, the use of calcium channel blockers (CCBs) was significantly more frequent in the control group than in the VR group (29.2% vs. 9.2%, *p* = 0.004). In addition, baseline thyroid-stimulating hormone (TSH) levels and the C-reactive protein-to-albumin (CRP/albumin) ratio were significantly higher in the control group than in the VR group (*p* = 0.03 and *p* = 0.007, respectively). No other statistically significant differences in baseline characteristics were observed between the groups (Table 1).

**Figure 2:**
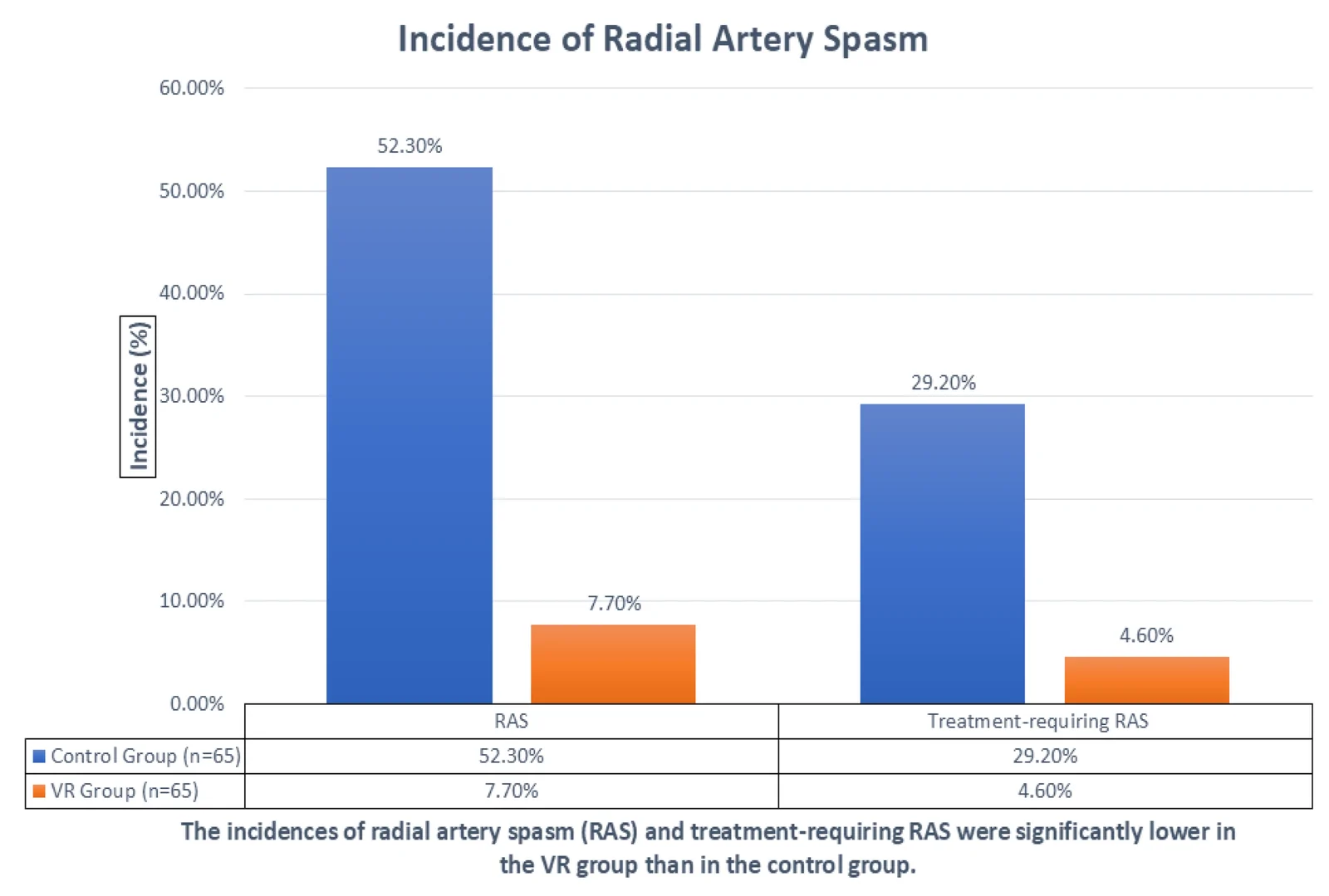
Incidence of radial artery spasm (RAS) and treatment-requiring RAS by study group. Both clinically assessed outcomes were less frequent in the VR group than in the control group.

Procedural characteristics, as well as the primary and secondary study endpoints, are presented in Table 2. The incidences of both overall RAS and treatment-requiring RAS were significantly lower in the VR group than in the control group (both *p* < 0.001; Figure 2). In addition, post-procedural STAI-S and Visual Analog Scale (VAS) scores were significantly lower in the VR group (both *p* < 0.001). Among the procedural variables, only contrast volume was significantly lower in the VR group (*p* = 0.02), whereas no significant between-group differences were observed for the remaining procedural parameters.

Compared with participants without RAS, those with RAS had higher contrast volume, more puncture attempts, higher post-procedural STAI-S scores, and higher VAS pain scores (all *p* < 0.01; Table 3). These post-procedural associations do not establish temporal prediction.

In the exploratory multivariable model, VAS score (OR, 3.44; 95% CI, 1.98–5.98; *p* < 0.001) and post-procedural STAI-S score (OR, 1.29; 95% CI, 1.13–1.47; *p* < 0.001) were associated with RAS. Contrast volume and puncture attempts were not independently associated (Table 4). Because pain and anxiety were recorded after sheath removal, these variables should not be interpreted as prospective predictors.

In the control group, the ΔSTAI-S score differed significantly between patients with and without RAS (*p* < 0.001), whereas this difference was not statistically significant in the VR group (*p* = 0.09). Among patients without RAS, ΔSTAI-S scores were higher than those of patients who developed RAS in both study groups (Table 5).

**Figure 3:**
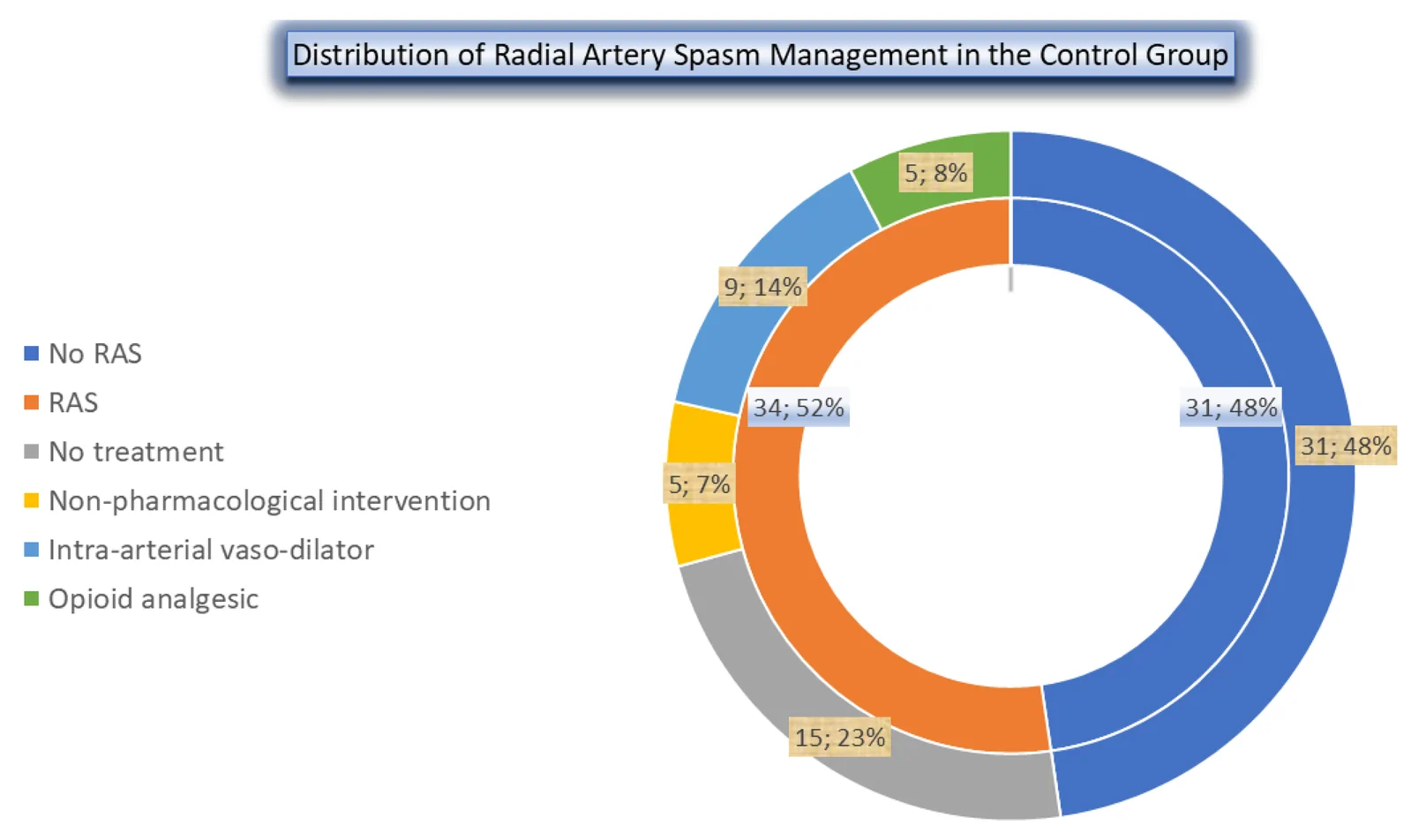
Distribution of RAS status and management strategies in the control group. The inner ring shows RAS status, and the outer ring shows management among participants with RAS.

Among patients who developed RAS, the need for treatment was comparable between the control and VR groups. Similarly, the distribution of treatment modalities, including non-pharmacological interventions, intra-arterial vasodilators, and opioid analgesics, was comparable between the two groups (Table 6). To complement this between-group comparison, Figure 3 provides a detailed overview of RAS occurrence and the distribution of management strategies within the control group (n = 65).

Because only five participants developed RAS in the VR group, with three requiring treatment, VR subgroup results are descriptive. In the control group, participants requiring treatment had higher post-procedural STAI-S scores and lower ΔSTAI-S scores than those not requiring treatment (both *p* < 0.001); the VAS difference was not significant (*p* = 0.14) (Table 7). These secondary analyses are exploratory and underpowered.

Only descriptive results are provided for treatment modalities in the VR group because three participants required treatment (Table 8). In the control group, post-procedural STAI-S did not differ across treatment modalities (*p* = 0.28), whereas ΔSTAI-S and VAS differed (*p* = 0.04 and *p* = 0.009). Post hoc findings are reported as exploratory: ΔSTAI-S differed between non-pharmacological and vasodilator groups (*p* = 0.047), and VAS was higher in the opioid group than in the non-pharmacological and vasodilator groups (*p* = 0.008 and *p* = 0.02).

## 4. Discussion

In this prospective randomized trial, VR allocation was associated with lower rates of clinically assessed RAS and treatment-requiring RAS and with lower post-procedural anxiety and pain in the per-protocol analysis. The magnitude of the RAS difference was large. It must be interpreted in light of the open-label design, subjective components of RAS ascertainment, absence of blinded objective corroboration, and exclusion of 44.9% of randomized participants from the final analysis. Post-procedural STAI-S and VAS were associated with RAS but cannot establish temporal prediction or mediation.

### 4.1. Effect of Virtual Reality on Anxiety

STAI-S decreased after TRA in both groups, with a larger observed reduction in the VR group. Post-procedural STAI-S was also lower with VR. These findings are consistent with an association between the intervention and lower measured anxiety, although expectancy, attention, and other open-label effects cannot be excluded.

Our findings are consistent with previous studies demonstrating the anxiety-reducing effects of VR. Recent meta-analyses have shown that VR-based interventions significantly reduce anxiety in patients undergoing surgery and invasive procedures [17,25,41]. Furthermore, favorable outcomes reported in cardiovascular interventions, including transcatheter aortic valve implantation and coronary angiography, support our findings [23,24]. Extending this evidence, the present study demonstrates that the use of VR during TRA is also associated with a significant reduction in procedural anxiety.

### 4.2. Effect of Virtual Reality on Pain

Participants receiving VR reported lower post-procedural VAS scores. This association is consistent with a distraction-related analgesic effect, but pain was measured after sheath removal and could also reflect procedural difficulty or RAS itself; causal direction cannot be determined.

Our findings are consistent with previous studies demonstrating the analgesic effects of VR. In addition to studies involving hospitalized patients and cardiovascular interventions, recent meta-analyses have shown that VR-based interventions significantly reduce procedure-related pain [25,41,42].

VAS and post-procedural STAI-S remained associated with RAS in the exploratory multivariable model. Because both were measured after the procedure, reverse causation is plausible: RAS, catheter resistance, manipulation, and treatment may have increased pain and anxiety. The model therefore cannot demonstrate that reductions in pain or anxiety mediated the observed difference in RAS.

### 4.3. Effect of Virtual Reality on Radial Artery Spasm

Ercan et al. reported an association between higher pre-procedural anxiety and RAS [11], supporting further study of psychological factors. In our trial, however, pre-procedural anxiety did not distinguish participants who later developed RAS, while post-procedural measures were temporally ambiguous. The lower RAS incidence observed with VR is therefore clinically interesting but does not by itself establish an anxiety-mediated mechanism.

Psychological stress can increase sympathetic activity and impair endothelial function [13,14], making a psychophysiological pathway plausible. Nevertheless, autonomic activity, endothelial function, and radial artery diameter were not measured, and no formal mediation analysis with temporally ordered variables was performed. Mechanistic interpretation remains hypothetical.

To date, studies evaluating the use of VR during cardiovascular interventions have primarily focused on patient comfort, anxiety, and pain control, consistently demonstrating beneficial effects on these psychological outcomes [24,25,43]. In contrast, evidence regarding its impact on procedure-related vascular complications remains limited. To the best of our knowledge, the present study is among the first prospective randomized studies to evaluate the effect of VR on the incidence of RAS during TRA.

If replicated with blinded or objective endpoint confirmation, VR could offer a non-invasive adjunct for patient comfort during TRA. Routine use specifically to prevent RAS cannot be recommended from this single-center per-protocol analysis alone.

### 4.4. Treatment-Requiring Radial Artery Spasm

RAS was diagnosed using prespecified clinical criteria, yielding an overall incidence of 30% [11]. Because clinical definitions and procedural practices vary [4,5,8,9,44], treatment-requiring RAS was examined separately. Treatment-requiring RAS was less frequent in the VR group (4.6% vs. 29.2%, *p* < 0.001), but this secondary finding is based on few events and remains exploratory.

Among patients in the control group, those who required treatment for RAS had higher post-procedural STAI-S scores and a smaller reduction in anxiety, as reflected by the ΔSTAI-S score. In contrast, although VAS pain scores were numerically higher in patients requiring treatment, the difference did not reach statistical significance. While the limited number of events precludes definitive conclusions, these findings suggest a potential association between higher procedural anxiety and the development of treatment-requiring RAS.

Previous studies have primarily focused on predictors of RAS occurrence, whereas data specifically addressing treatment-requiring RAS remain scarce [5,8,9,10]. Our findings highlight the importance of evaluating treatment-requiring RAS as a distinct clinical outcome. Larger multicenter studies are warranted to validate these findings and to further clarify the effect of VR on treatment-requiring RAS.

### 4.5. Association Between Anxiety and the Development of Radial Artery Spasm

Post-procedural anxiety was higher among participants with RAS, whereas baseline anxiety was similar. This pattern should not be interpreted as evidence that anxiety caused RAS: RAS-related pain, manipulation, and treatment may instead have increased the post-procedural score. Only repeated measurements obtained before and during RAS onset could establish temporal ordering.

In the control group, ΔSTAI-S was lower among participants with RAS. Because ΔSTAI-S incorporates a post-procedural value measured after the event, the association is compatible with both directions of effect. The five RAS events in the VR group precluded reliable inference, and no mediation conclusion is supported.

Similarly, lower ΔSTAI-S scores among patients with treatment-requiring RAS suggest that an insufficient reduction in anxiety may be associated with the development of RAS requiring additional pharmacological or non-pharmacological intervention. However, these findings regarding ΔSTAI-S are based on subgroup comparisons and were not confirmed in the multivariable analysis.

### 4.6. Evaluation of Additional Clinical and Procedural Findings

CCBs may reduce radial vasoconstriction, and oral CCB use was imbalanced despite randomization (control 29.2% vs. VR 9.2%, *p* = 0.004) [4,6,7,32,33,34,45]. The direction of this imbalance would be expected to favor the control group, yet RAS was more frequent in that group. Nevertheless, baseline imbalance remains a potential confounder in this modest sample, especially because oral agent, dose, adherence, and indication were not incorporated into the primary treatment comparison.

Contrast volume was significantly higher in the control group and was associated with RAS development in the univariable analysis. However, this association was no longer significant after adjustment in the multivariable model. These findings suggest that contrast volume may not represent an independent determinant of RAS but rather a secondary marker reflecting the increased contrast requirement resulting from more difficult catheter manipulation and prolonged procedural time caused by the development of radial artery spasm.

Similarly, although the number of puncture attempts was associated with RAS development in the univariable analysis, it was not identified as an independent predictor in the multivariable analysis. Previous studies have suggested that repeated puncture attempts may contribute to the development of RAS by increasing local vascular trauma and procedural pain [5,8,9,10]. However, it is also plausible that the occurrence of RAS itself makes arterial access and catheter manipulation more challenging, thereby increasing the need for additional puncture attempts. Therefore, a higher number of puncture attempts may represent a consequence of procedural difficulty secondary to RAS rather than a causal factor in its development.

Taken together, these findings indicate that the development of RAS is a multifactorial process that cannot be explained by any single clinical, biochemical, or procedural parameter. CCB use, contrast volume, and the number of puncture attempts alone were insufficient to account for the occurrence of RAS. Instead, our results support the concept that RAS arises from the complex interplay of patient characteristics, psychological factors, vascular anatomy, procedural factors, and operator experience.

### 4.7. Study Limitations

This study has important limitations. It was conducted at one center with a modest sample. Participants and the operator could not be blinded, and the large observed effect is susceptible to performance and detection bias. Although allocation was concealed and the statistician was blinded, RAS was assessed by the unblinded operator using clinical criteria that included subjective pain and resistance; there was no blinded adjudication, angiographic endpoint, or ultrasound corroboration.

The final analysis excluded 106 of 236 randomized participants (44.9%), predominantly after PCI, and was therefore per-protocol rather than intention-to-treat. Even though exclusions followed prespecified criteria and were balanced numerically by group, post-randomization selection can disrupt comparability, introduce attrition bias, and limit applicability to the broader population initially randomized.

Pain and anxiety were self-reported after sheath removal, in the same general period in which RAS occurred and could have been treated. Reverse causation is therefore plausible, and the regression cannot distinguish whether anxiety/pain preceded, accompanied, or followed RAS. Baseline CCB use, TSH, and CRP/albumin ratio were imbalanced; residual confounding remains possible. The study used only a 6 Fr system and included elective TRA, limiting extrapolation to 5 Fr systems and acute coronary syndromes.

Finally, few treatment-requiring RAS events—especially in the VR group—made secondary and treatment-stratified analyses unstable and hypothesis-generating. Headset removals were not characterized by specific symptoms, and cost, workflow burden, patient preference, and cost-effectiveness were not assessed. Future multicenter trials should prespecify intention-to-treat analysis, use objective or blinded RAS confirmation, capture time-resolved anxiety and pain before RAS onset, compare sheath sizes, systematically record tolerability, and include implementation and cost-effectiveness outcomes.

## 5. Conclusions

In this single-center per-protocol analysis, VR use during TRA was associated with lower post-procedural anxiety and pain and a lower incidence of clinically assessed RAS. The large effect estimate is promising but not definitive because blinding was infeasible, the primary endpoint contained subjective elements, and substantial post-randomization exclusions occurred. Larger multicenter intention-to-treat trials with blinded or objective RAS confirmation are needed before VR can be recommended as a routine strategy to prevent RAS.

## Figures and Tables

**Table 1:** Baseline characteristics of the study population.

Variable	Control (n = 65)	VR (n = 65)	*p* Value
**Demographic Characteristics**			
Age, years	58.6 ± 11.0	57.1 ± 9.8	0.41 †
Female sex, n (%) Male sex, n (%)	34 (52.3) 31 (47.7)	28 (43.1) 37 (56.9)	0.29 §
BMI, kg/m^2^	30.20 ± 5.08	28.96 ± 4.44	0.10 †
BSA, mean ± SD	1.93 ± 0.2	1.95 ± 0.19	0.57 †
**Cardiovascular Risk Factors**			
Current smoking, n (%)	30 (46.2)	24 (36.9)	0.29 §
Alcohol use, n (%)	2(3.1)	5 (7.7)	0.24 §
Diabetes mellitus, n (%)	22 (33.8)	18 (27.7)	0.45 §
Hypertension, n (%)	37 (56.9)	25 (38.5)	0.08 §
**Baseline Medications**			
ASA, n (%)	42 (64.6)	35 (53.8)	0.21 §
P2Y12I, n (%)	5 (7.7)	2 (3.1)	0.44 §
RAAS Inhibitor, n (%)	25 (38.5)	20 (30.8)	0.36 §
Beta-Blocker, n (%)	27 (41.5)	21 (32.3)	0.28 §
Statin, n (%)	20 (30.8)	25 (38.5)	0.36 §
Nitrate, n (%)	3 (4.6)	1 (1.5)	0.31 §
Ranolazine, n (%)	0 (0)	1 (1.5)	0.32 §
CCB, n (%)	19 (29.2)	6 (9.2)	**0.004 §**
Oral Antidiabetic Medication, n (%)	17 (26.2)	15(23.1)	0.68 §
Insulin, n (%)	4 (6.2)	2 (3.1)	0.34 §
**Laboratory Parameters**			
Creatinine, mean ± SD	0.81 ± 0.18	0.81 ± 0.16	0.99 †
eGFR, mean ± SD	91.23 ± 16.42	91.38 ± 13.44	0.96 †
ALT, median (IQR)	20 (17–26.5)	21 (15–31)	0.87 ‡
Albumin, mean ± SD	40.19 ± 7.28	39.39 ± 5.71	0.49 †
CRP, median (IQR)	3.30 (1.5–13.4)	2.1 (1–4.6)	0.08 ‡
LDL Cholesterol, mean ± SD	134.91 ± 30.02	127.81 ± 36.39	0.2 †
TSH, median (IQR)	1.65 (1.2–2.23)	1.22 (1–1.97)	**0.03 ‡**
Hemoglobin, mean ± SD	13.77 ± 1.67	13.75 ± 1.66	0.94 †
CRP-to-Albumin Ratio, median (IQR)	0.09 (0.05–0.15)	0.05 (0.02–0.11)	**0.007 ‡**
**Echocardiographic Parameters**			
LVEF, mean ± SD	56.55 ± 7.61	56.29 ± 6.96	0.84 †

† Student’s *t* test; ‡ Mann–Whitney *U* test; § Chi-square test. A two-sided *p* value < 0.05 was considered statistically significant. BMI, body mass index; BSA, body surface area; CCB, calcium channel blocker; ASA, acetylsalicylic acid; RAAS, renin–angiotensin–aldosterone system; CRP, C-reactive protein; TSH, thyroid-stimulating hormone; LDL-C, low-density lipoprotein cholesterol; LVEF, left ventricular ejection fraction; eGFR, estimated glomerular filtration rate; ALT, alanine aminotransferase.

**Table 2:** Procedural Characteristics and Study Outcomes According to Study Group.

Variable	Control (n = 65)	VR (n = 65)	*p* Value
**Procedural Parameters**			
Number of catheters used, n (%) 2 3	59 (90.8) 6 (9.2)	61 (93.8) 4 (6.2)	0.51 ¶
Number of puncture attempts, median (IQR)	2 (1–2)	2 (1–2)	0.40 ‡
Radiation dose, median (IQR)	352 (207–446)	280 (178.5–393.5)	0.11 ‡
Fluoroscopy time (s), median (IQR)	150 (85–210)	120 (75–167)	0.14 ‡
Procedure duration (min), mean ± SD	11.68 ± 4.2	9.43 ± 3.78	0.98 †
Contrast volume (mL), mean ± SD	57.79 ± 18.73	49.31 ± 14.49	**0.02 †**
**Anxiety, Pain, and Radial Artery Spasm**			
Pre-procedural STAI-S, mean ± SD	40.29 ± 11.58	39.12 ± 10.48	0.58 †
Post-procedural STAI-S, mean ± SD	34.58 ± 10.89	27.74 ± 7.98	**<0.001 †**
***p* value (within group)**	**0.003 §**	**<0.001 §**	
VAS score, median (IQR)	5 (2–6)	4 (2–5)	**<0.001 ‡**
RAS, n (%)	34 (52.3)	5 (7.7)	**<0.001 ¶**
**Treatment-requiring RAS, n (%)**	19 (29.2)	3 (4.6)	**<0.001 ¶**

† Student’s *t* test; ‡ Mann–Whitney *U* test; § Paired *t* test; ¶ Chi-square test. *p* < 0.05 was considered statistically significant. Abbreviations: VAS, Visual Analog Scale; RAS, radial artery spasm; STAI-S, State Anxiety.

**Table 3:** Comparison of patients with and without radial artery spasm.

Characteristics	No RAS n = 91	RAS n = 39	*p* Value
Age (years), mean ± SD	58.3 ± 10.2	56.7 ± 10.9	0.43 †
Body mass index (BMI), mean ± SD	29.55 ± 4.87	29.55 ± 4.49	0.99 †
Sex, n (%)			0.36 §
Female	41 (45.1)	21 (53.8)	
Male	50 (54.9)	18 (46.2)	
TSH, median (Q1–Q3)	1.48 (1.01–2.12)	1.5 (1.06–1.99)	0.85 ‡
CRP/Albumin ratio, median (Q1–Q3)	0.06 (0.03–0.13)	0.08 (0.05–0.16)	0.15 ‡
Contrast volume used, mean ± SD	49.29 ± 11.81	63.46 ± 16.63	**0.003 †**
Number of puncture attempts, median (Q1–Q3)	1 (1–2)	2 (2–3)	**<0.001 ‡**
VAS score, median (Q1–Q3)	2 (1–4)	6 (5–8)	**<0.001 ‡**
Pre-procedural STAI-S score, mean ± SD	39.16 ± 11.06	40.97 ± 11.31	0.44 †
Post-procedural STAI-S score, mean ± SD	27.25 ± 5.94	40.28 ± 8.79	**<0.001 †**

† Student’s *t* test; ‡ Mann–Whitney *U* test; § Chi-square test. Statistical significance was defined as *p* < 0.05. Abbreviations: BMI, body mass index; TSH, thyroid-stimulating hormone; CRP, C-reactive protein; VAS, visual analog scale; STAI-S, State Anxiety; RAS, radial artery spasm.

**Table 4:** Exploratory multivariable logistic regression analysis of factors associated with RAS.

Exposure Variables	Odds Ratio	95% CI: Lower-Upper	Regression Coefficient *p*-Value
Contrast volume used	1.01	0.98–1.04	0.52
Number of puncture attempts	0.99	0.46–2.12	0.98
VAS	3.44	1.98–5.98	**<0.001**
Post-procedural STAI-S score	1.29	1.13–1.47	**<0.001**

Abbreviations: CI, confidence interval; RAS, radial artery spasm; VAS, visual analog scale; STAI-S, State Anxiety Inventory–State score.

**Table 5:** Change in state anxiety (ΔSTAI-S) according to study group and RAS status.

ΔSTAI-S	Control Group n = 65	VR Group n = 65
RAS status	Median (Q1–Q3)	Median (Q1–Q3)
RAS developed (n:34/5)	−4.5 (−9.25 to 12.25)	−9 (−15 to 15)
No RAS (n:31/60)	7 (4 to 19)	10 (7 to 19.5)
*p* value	**<0.001**	0.09

Statistical significance was defined as *p* < 0.05. Abbreviations: RAS, radial artery spasm; VR, virtual reality; ΔSTAI-S, change in State Anxiety Inventory score.

**Table 6:** Comparison of treatment requirements and treatment modalities according to VR use among patients who developed radial artery spasm.

Patients with RAS (n = 39)	Control Group n = 34 (%)	VR Group n = 5 (%)
**Treatment requirement**		
No treatment	15 (44.1)	2 (40)
Treatment required	19 (55.9)	3 (60)
**Treatment modality**		
Non-pharmacological intervention	5 (26.3)	2 (66.7)
Intra-arterial vasodilator	9 (47.4)	1 (33.3)
Opioid analgesic	5 (26.3)	0 (0)

Abbreviations: RAS, radial artery spasm; VR, virtual reality.

**Table 7:** Comparison of post-procedural anxiety and pain scores according to virtual reality use and treatment requirement among patients who developed radial artery spasm.

**Patients with RAS in the VR Group (n = 5)**	**No Treatment Required n = 2**	**Treatment Required n = 3**	***p*-Value**
Post-procedural STAI-S score, mean ± SD	33.5 ± 4.9	44.3 ± 8.5	-
ΔSTAI-S score, mean ± SD	15 ± 1.4	−13 ± 6.1	-
VAS score, median (IQR)	6.5 (5–8)	7 (6–7.5)	-
**Patients with RAS in the Control Group (n = 34)**	**No treatment Required n = 15**	**Treatment Required n = 19**	***p*-Value**
Post-procedural STAI-S score, mean ± SD	32.5 ± 4.9	46.5 ± 8.6	<0.001 *
ΔSTAI-S score, mean ± SD	13.5 ± 7.2	−8.7 ± 6.4	<0.001 *
VAS score, median (IQR)	5 (4–6)	6 (5–8)	0.14 †

* Student’s *t* test; † Mann–Whitney *U* test. *p* < 0.05 was considered statistically significant. Abbreviations: RAS, radial artery spasm; VR, virtual reality; ΔSTAI-S, change in State Anxiety Inventory score; VAS, visual analog scale; STAI-S, State Anxiety.

**Table 8:** Comparison of Post-Procedural STAI-S, Change in STAI-S Score, and VAS According to Treatment Modality in Patients Requiring Treatment for Radial Artery Spasm.

**VR Group Requiring Treatment for RAS (n = 3)**	**Non-Pharmacological** **n = 2**	**Vasodilator** **n = 1**	**Opioid** **n = 0**	***p*-Value**
Post-procedural STAI-S, mean ± SD	39 ± 7.1	55 ± -	-	-
ΔSTAI-S score, mean ± SD	−9.5 ± 0.7	−20 ± -	-	-
VAS Score, median (Q1–Q3)	6	6.5	-	-
**Control Group Requiring Treatment for RAS (n = 19)**	**Non-Pharmacological** **n = 5**	**Vasodilator** **n = 9**	**Opioid** **n = 5**	***p*-Value**
Post-procedural STAI-S, mean ± SD	40 ± 12.4	48.2 ± 10.4	50 ± 8.1	0.28 *
ΔSTAI-S score, mean ± SD	−1.8 ± 4.7	−11.3 ± 3.4	−11 ± 8.1	0.04 *
VAS Score, median (Q1–Q3)	5 (4.5–5.5)	6 (4.5–8)	8 (7–9)	**0.009 †**

* One-way ANOVA test; † Kruskal–Wallis H test. *p* < 0.05 was considered statistically significant. **Abbreviations:** RAS, radial artery spasm; VR, virtual reality; ΔSTAI-S, change in State Anxiety Inventory score; VAS, visual analog scale; STAI-S, State Anxiety.

## Data Availability

The data presented in this study are available from the corresponding author upon reasonable request. The data are not publicly available due to privacy and ethical restrictions.

## Supplementary Materials

The following supporting information can be downloaded at: https://www.mdpi.com/article/10.3390/jcm15187227/s1, Figure S1. Schematic illustration of the virtual reality setup during transradial coronary angiography.
