# Supplementary material for: Effect of Virtual Reality on Anxiety, Pain, and Radial Artery Spasm During Transradial Coronary Angiography: A Prospective Randomized Controlled Trial
Source: J Clin Med. 2026 Sep 17;15(18):7227. doi: 10.3390/jcm15187227 (PMC13607304; doi:10.3390/jcm15187227)
Supplement: Supplementary file 1 [file jcm-15-07227-s001.zip › jcm-4506826-supplementary.pdf]

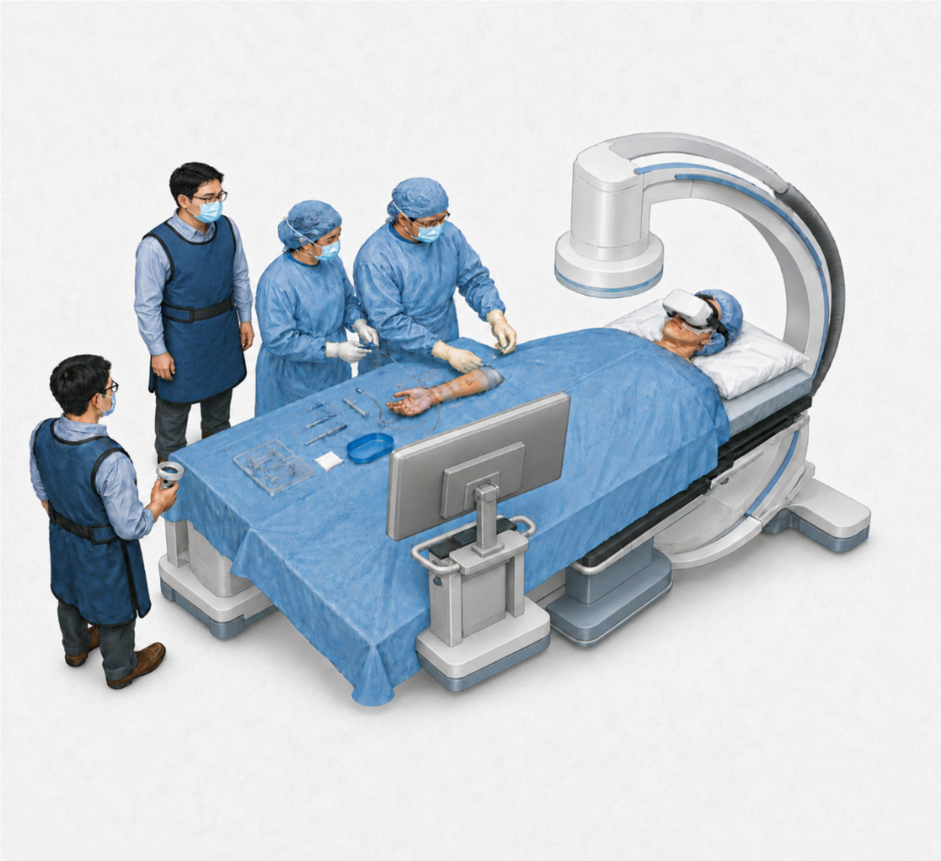

Supplementary Figure S1. Schematic illustration of the virtual reality setup during transradial coronary angiography. The illustration depicts the positioning of the patient, healthcare personnel, VR headset, angiography monitor, and sterile field during the procedure. It does not represent an actual study participant or procedure. The illustration was generated using OpenAI's ChatGPT and subsequently reviewed and revised by the authors for scientific accuracy.
